# Single botanical drugs in the Ayurvedic Pharmacopoeia of India—A quantitative ethnobotanical analysis

**DOI:** 10.3389/fphar.2023.1136446

**Published:** 2023-05-11

**Authors:** Ruyu Yao, Michael Heinrich, Bengang Zhang, Xueping Wei, Yaodong Qi, Weiwei Gao

**Affiliations:** ^1^ Institute of Medicinal Plant Development, Chinese Academy of Medical Sciences and Peking Union Medical College, Beijing, China; ^2^ Research Group “Pharmacognosy and Phytotherapy”, UCL School of Pharmacy, University of London, London, United Kingdom

**Keywords:** botanical drug, medical flora, Pharmacopoeia, ayurveda, quantitative ethnobotany

## Abstract

Developing evidence-based uses of herbal medicines and natural product-based drug discovery are two core aims of ethnopharmacology. This requires an understanding of the medicinal plants and the traditional medical knowledge associated with them which is a basis for cross-cultural comparison. The botanical drugs of traditional medical systems are still not understood well, even for well-known and widely respected traditions like Ayurveda. In this study, a quantitative ethnobotanical analysis was performed on the single botanical drugs included in the Ayurvedic Pharmacopoeia of India (API), presenting an overview on the medicinal plants of Ayurveda from perspectives of plant systematics and medical ethnobotany. Part-I of API includes 621 single botanical drugs, which are sourced from 393 species (323 genera in 115 families). Of these, 96 species yield two or more drugs, together accounting for 238 drugs. Taking the traditional concepts, biomedical uses and the pragmatic disease classification into account, therapeutic uses of these botanical drugs are sorted into 20 categories, which meet primary health demands. The therapeutic uses of the drugs sourced from the same species may differ considerably, but 30 of the 238 drugs are used in highly similar way. The comparative phylogenetic analysis identifies 172 species with high potential for specific therapeutic uses. This medical ethnobotanical assessment for the first time provides a comprehensive understanding on the single botanical drugs in API from the perspective of medical botany using an “etic” (scientist-oriented) approach. This study also highlights the importance of quantitative ethnobotanic methods in understanding traditional medical knowledge.

## Introduction

Botanical drugs are the most common medical resources used in traditional medical systems, have played a key role in primary healthcare and are still the main sources for new drug discovery (Heinrich and Jäger, 2015; Newman and Cragg, 2020; Atanasov et al., 2021). Single botanical drugs, the botanical drugs sourced from a single plant, are the basic traditional medical materials. The medical usages of one plant are generally based on diverse factors, thus, one given medicinal species will often be used for several purposes, which complicate the evidence-based uses of herbal medicines and natural product-based drug discovery. Consequently, the historical/traditional uses of botanical drugs provide important clues for new drug discovery and are a core basis for their legal acceptance by regulatory agencies (Jutte et al., 2017; Lenssen et al., 2019). However, the regional knowledge on botanical drugs is often interpreted with heterogenous terms, which may lead to inadequate uses or even the misidentification of the plant species especially in a cross-cultural environment (Rivera et al., 2014; Yao et al., 2021; Heinrich et al., 2022b). A comprehensive understanding the botanical drugs of a medical flora, the botanical drugs used within a tradition, and the traditional and local knowledge associated with this is critical for developing evidence-based uses of herbal medicines and natural product-based drug discovery.

Today 65% of people in rural India still rely on traditional medicines (Sen and Chakraborty, 2015), especially using botanical drugs derived from Ayurveda, but also Siddha and Unani traditions. Globally, Ayurveda is among the most popular traditional medicinal practices. It originated in South Asia and has been known since the Vedic era (ca. 1500 to 600 BCE), as evidenced in the ancient literature such as Charaka Samhita and Sushruta Samhita (National Health Portal of India, 2016; Jaiswal and Williams, 2017). Ayurvedic medical interventions rely on botanical drugs, with approximately ninety percent of its preparations being plant-based, and eighty percent of the Ayurvedic medicines being sourced from plants (Joshi et al., 2017; Kumar et al., 2017). Approximately 7,500 plant species are used medicinally in India, and 2,000 of those are used in Ayurveda, with 700 being used frequently (Sen et al., 2011; Pan et al., 2014). Since 1989, part I of the Ayurvedic Pharmacopoeia of India (API) has published nine volumes (the last one in 2016). It is a crucial reference for the identification and quality control of single drugs used in Ayurveda (Joshi et al., 2017). Part II of API is for the medicated formulations with multi-ingredients, so we did not include it in the present study on single botanical drugs. Previously, Jia et al. (2012) have described the single drugs in the first 7 volumes of part I of API, while Jaiswal et al. (2016) discussed the different usages and processing of selected single botanical drugs in API and China. The above studies have provided an initial understanding on the single botanical drugs in API. However, a systematic scientific understanding on these drugs is still not available, which limits their broader cross-cultural use.

Plants with close phylogenetic relationship are likely to be similar in their metabolome (i.e., share classes of metabolites) and often have similar traditional uses (Zaman et al., 2021). In practice, this complexity might be overlooked in scientific processes which do not encompass an approach capturing a species chemical complexity. One option to overcome this is through comparative phylogeny, which enables the identification of taxa with high potential for specific therapeutic uses, as indicated by phylogenetic clustering (Lei et al., 2020; Zaman et al., 2021). In the present study, the single botanical drugs in API are studied systematically using a quantitative ethnobotanic approach. Moreover, borrowing methods of comparative phylogeny, taxa with high potential for specific therapeutic uses are suggested.

## Materials and methods

### Data collection

Data of the single botanical drugs were collected from the Part I of API, including 9 volumes as shown in Table 1. For each of the herbal monographs, information on the herbal name, biological origin(s), medicinal plant-part(s), therapeutic use(s) were extracted. The scientific names of the biological origins were then checked against World Flora Online (WFO) using R package “U.Taxonstand” (Zhang and Qian, 2022) with R 4.2.0 (R Core Team, 2022). Since the therapeutic uses of the herbal drugs in volume one to five were only available in Latinized Sanskrit, these terms were translated as follows: 1) to build a Sanskrit-English dictionary with the therapeutic use data in volume six to nine; 2) to translate the therapeutic uses into English with the above dictionary; 3) for those terms which were not included in the above dictionary, search in Wisdom Library (https://www.wisdomlib.org/) manually and cross-checked with the standardization of non-clinical terminologies of ayurveda (National Institute of Ayurveda, 2011). As a result, data including the drug names, biological origins, medicinal plant-parts, as well as the therapeutic uses of all the single botanical drugs in API were compiled (Supplementary Materials S1, S2).

**TABLE 1:** The 9 volumes of the Ayurvedic Pharmacopoeia of India (part I).

Volume	Contents	References
1	80 monographs for single drugs	Ministry of HFW (1986)
2	78 monographs for single drugs	Ministry of HFW (1999)
3	100 monographs for single drugs	Ministry of HFW (2001)
4	68 monographs for single drugs	Ministry of HFW (2004)
5	92 monographs for single drugs	Ministry of HFW (2006)
6	101 monographs for single drugs	Ministry of HFW (2008a)
7	21 monographs for single drugs (minerals or metals)	Ministry of HFW (2008b)
8	60 monographs, for 15 single drugs and their extracts	Ministry of HFW (2011)
9	45 monographs, for 15 single drugs and their extracts	Ministry of AYUSH (2016)

### Therapeutic use categorization

While understanding traditional practice clearly and essential aim of any ethnopharmacological study, there now is a long tradition of using an etic categorization in order to allow an understanding of biomedical uses and the future potential of these species (Staub et al., 2015). In anthropological terms “etic” approaches (borrowed from the term phonetic in linguistics) investigate elements of a culture from an outsider’s perspective focusing on cross-cultural differences and similarities. Since it provides a global standard, it also enables a comparative analysis (Yao et al., 2021). Additionally, we suggest that the categorization should also consider the traditional concept and the pragmatic category in modern hospital. Finally, these therapeutic uses were sorted into 20 categories, including AND (andrology and male reproductive system), ANT (antidote and problems due to poisoning), CAR (cardiovascular disorders, blood-related diseases), DER (dermatologic disorders, skin problems), EAR (ear and hearing problems), EYE (eye and vision disorders), GAS (gastrointestinal and digestive disorders), GYN (gynecology and female reproductive system), GNR (general complaints including cold, fever, inflammation, headache, etc*.*), IMM (immune disorders and rheumatism), MET (metabolic and endocrine disorders), NER (nervous system, memory and sleeping problems), ORA (oral and dental problems), PAR (parasitic disease), PED (pediatrics disorders), PSY (psychiatric disorders and mental illness), RES (respiratory system disorders), SKE (skeleto-muscular system disorders), TRA (traditional uses that were not related to modern indications), URO (urinary disorders).

### Data analysis

To generate a phylogenetic tree of all species, R package “V.PhyloMaker2” (Jin and Qian, 2022) was used with R 4.2.0 (R Core Team, 2022). And then, with formatted tables (i.e., “sample” and “traits”) generated from the data obtained above, this phylogenetic tree was loaded into Phylocom 4.2 (Webb et al., 2008). According to the manual, the following indices for each therapeutic use category were calculated: Faith’s phylogenetic diversity (PD), diversity weighted by interspecific phylogenetic distances (Dp), mean pairwise trait distance among taxa (MPD), net relatedness index (NRI), mean distance to nearest neighbor trait distance (MNTD) and nearest taxon index (NTI). Additionally, “hot nodes,” which mean the nodes with significantly more taxa than random probability for specific therapeutic uses were detected as marked with “SIGMORE” (Webb et al., 2008). Data visualization was performed using iTOL (Letunic and Bork, 2021).

To evaluate the therapeutic similarity of drugs sourced from the same species, the pairwise Jaccard Index (JI) was calculated based on their categorized therapeutic uses data, using the R package “sets.” And then, those JI of the same species were selected manually (as highlighted in Supplementary Material S3), thereafter, a distribution histogram of these JI values were plotted with interval of 0.1 was plotted using Microsoft Excel 365. A Cullen and Frey graph was then mapped to test the distribution pattern using R package “fitdistrplus” (Delignette-Muller & Dutang, 2015) with R 4.2.0 (R Core Team, 2022).

## Results

### An overview of the single drugs in API

Part I of API consists of 645 monographs for single drugs or extracts, and almost all of these are single botanical drugs except the following 27: 1) Volume VII records 21 drugs sourced exclusively from minerals or metals; 2) volume VI includes three non-botanical drugs (i.e., butter, water and honey; 3) volume VI includes two drugs which are the mixture of plant extracts from two families thus they are not “single”; 4) volume III includes one drug which is the fungal part of a lichen. Besides, different parts of three botanical drugs were with distinguishable therapeutic uses, so these different parts were treated as individual botanical drugs. As a result, 621 single botanical drugs were identified in API in total (A list of these 621 drugs is provided in Supplementary Material S1).

### Taxonomic diversity of biological sources for the single botanical drugs in API

**FIGURE 1:**
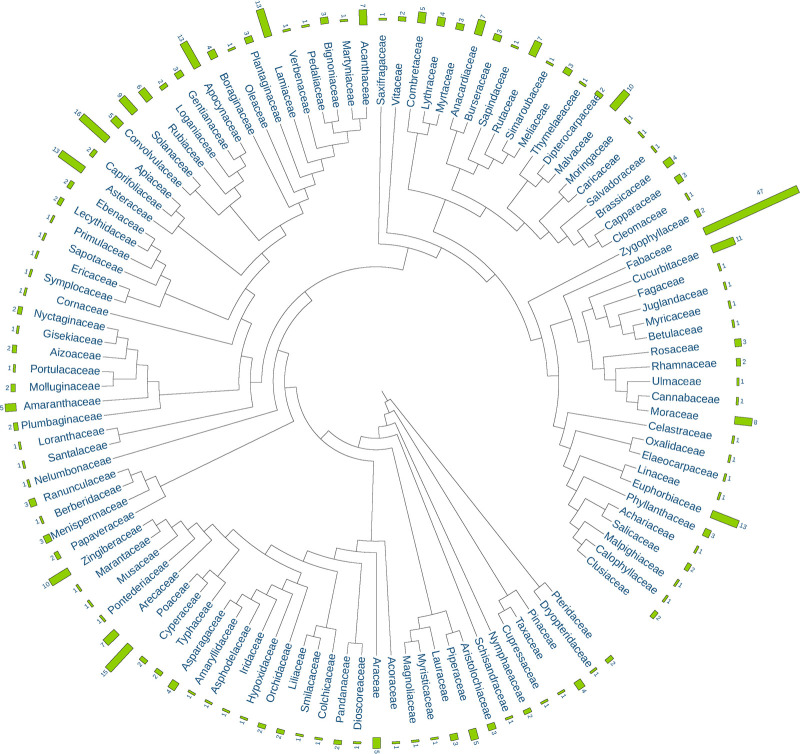
Composition of plant families and species for single botanical drugs in the Ayurvedic Pharmacopoeia of India (API).

The 621 single botanical drugs in API are sourced from 393 species in 323 genera and 115 families based on APG IV—the current Angiosperm Phylogeny Group’s classification (Figure 1). With 47 species, Fabaceae is the largest source family of these botanical drugs, which is followed by Apiaceae (16), Poaceae (15), Euphorbiaceae (13), Lamiaceae (13), Asteraceae (13), Apocynaceae (13), Cucurbitaceae (11), Malvaceae (10), Zingiberaceae (10), Solanaceae (9), and Moraceae (8). The top 12 families together contribute 178 species which constitute 45.3% of the total source species. Other families such as Acanthaceae, Arecaceae, Anacardiaceae, Rubiaceae, Amaranthaceae, Araceae, Combretaceae, Convolvulaceae and Piperaceae contribute less species to the Ayurvedic medical flora.

As expected, some large families with distinct species like the Fabaceae, Euphorbiaceae, Lamiaceae and Asteraceae are commonly included, but some with a less distinct characteristics like the Poaceae and some smaller but very characteristic families like the Zingiberaceae are also present. It is worth noting that as many as 75 families contribute only one or two species, and these families have increased the phylogenetic diversity of the Ayurvedic medical flora largely.

### Medicinal plant-parts of the single botanical drugs in API

**FIGURE 2:**
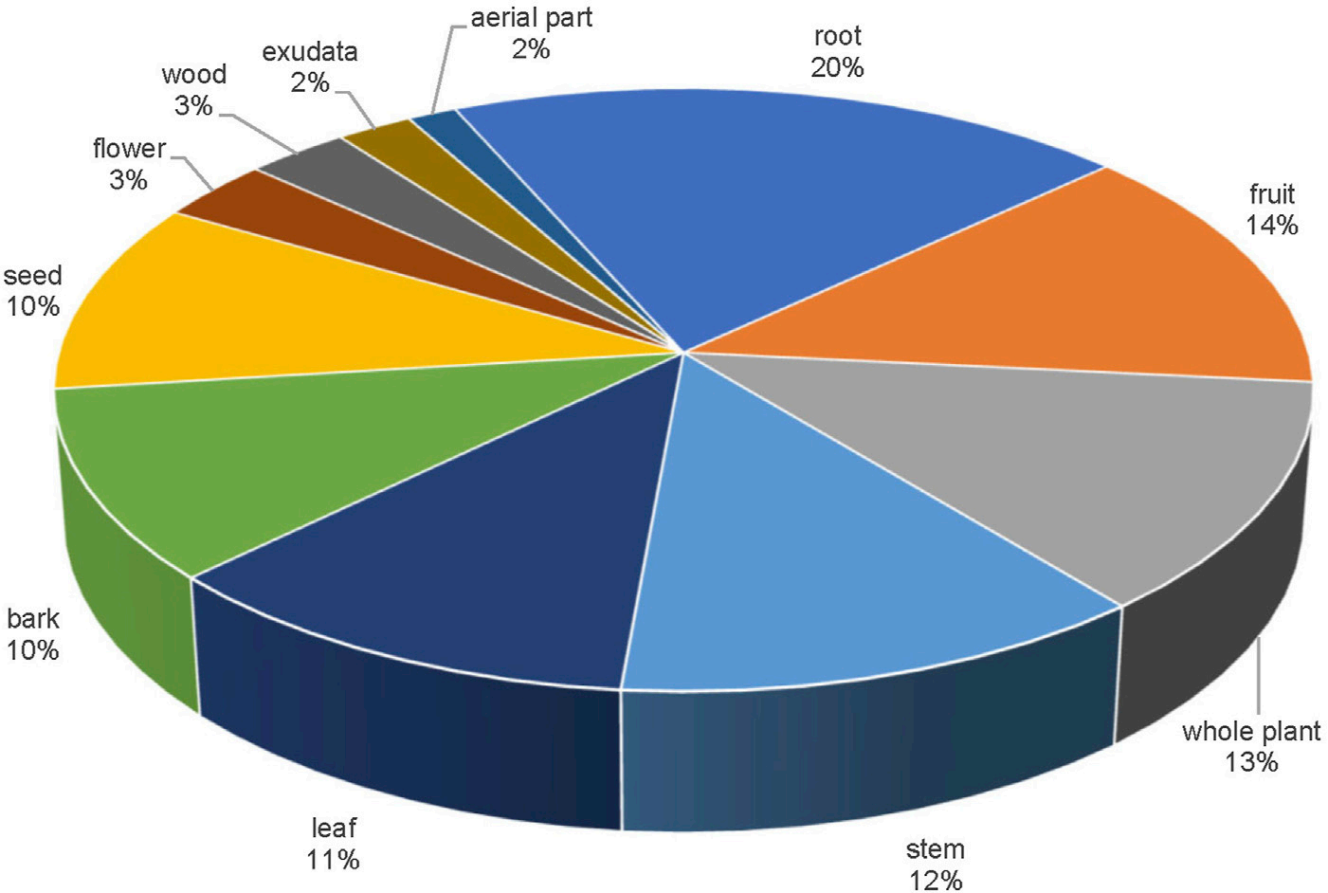
Constitution of medicinal parts for the single botanical drugs in API.

In terms of the medicinal part, the 621 single botanical drugs (Figure 2) include125 roots, (20%) making it the most commonly used medicinal part. Fruit rank second (85 drugs; 14%). These are followed by whole plant (13%), stem (12%), leaf (11%), bark (10%), and seed (10%).

### Therapeutic uses of the single botanical drugs in API

**FIGURE 3:**
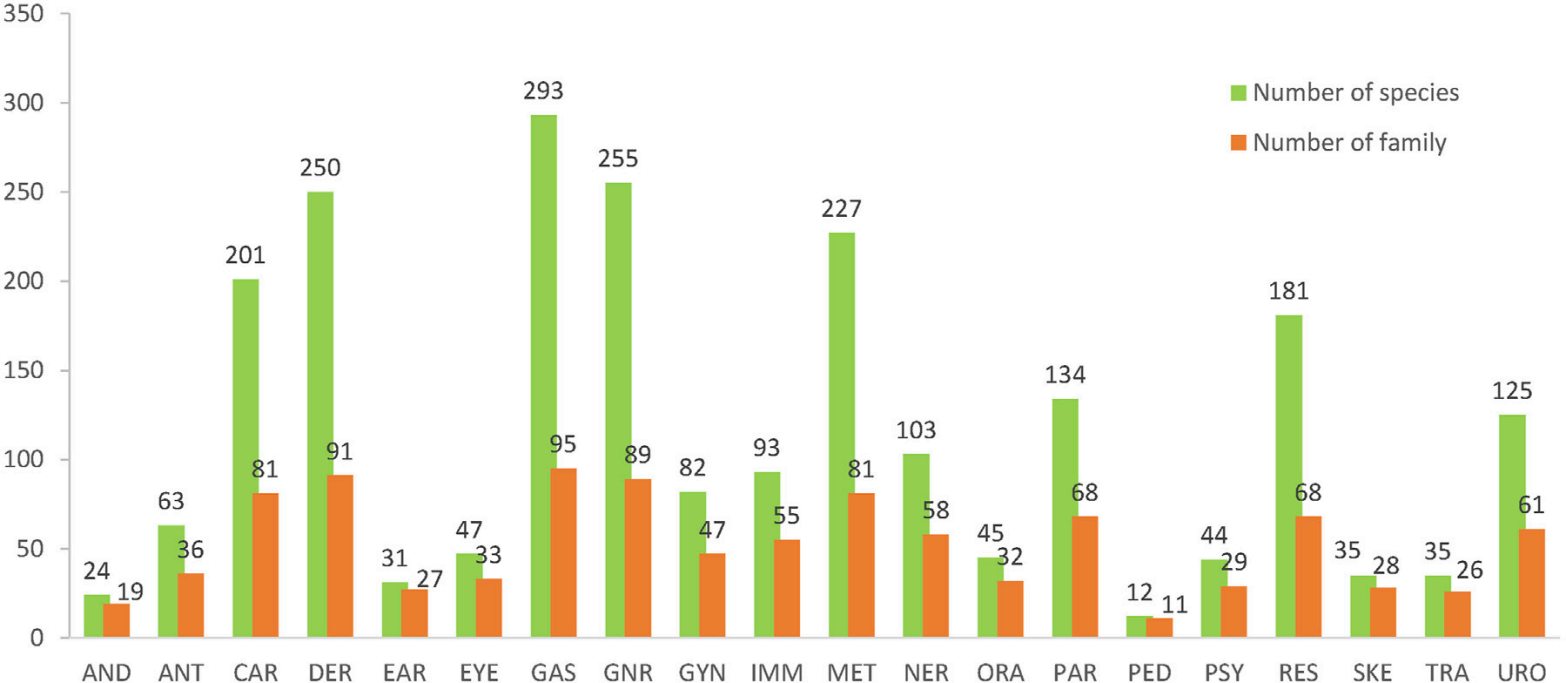
The numbers of species/families used for the therapeutic uses in API.

Taking the traditional concepts, biomedical uses and the pragmatic disease classification into account, therapeutic uses of the botanical drugs were sorted into 20 categories (Figure 3 **)**. There are 293 species of 95 families used in GAS (75% of all species and 82% of all families). This is followed by that of GNR (255 species, 89 families), DER (250 species, 91 families), MET (227 species, 81 families), CAR (201 species, 81 families), RES (181 species, 68 families), PAR (134 species, 58 families), and URO (125 species, 61 families). Interestingly, the above-mentioned therapeutic uses, which are linked to more species or families than the rest, are with a higher average species per family, ranging from 3.1 to 2.0; conversely, for those uses linking to less species and families, the average species per family are below 2.0.

### The similarity in therapeutic uses of drugs sourced from the same species

**FIGURE 4:**
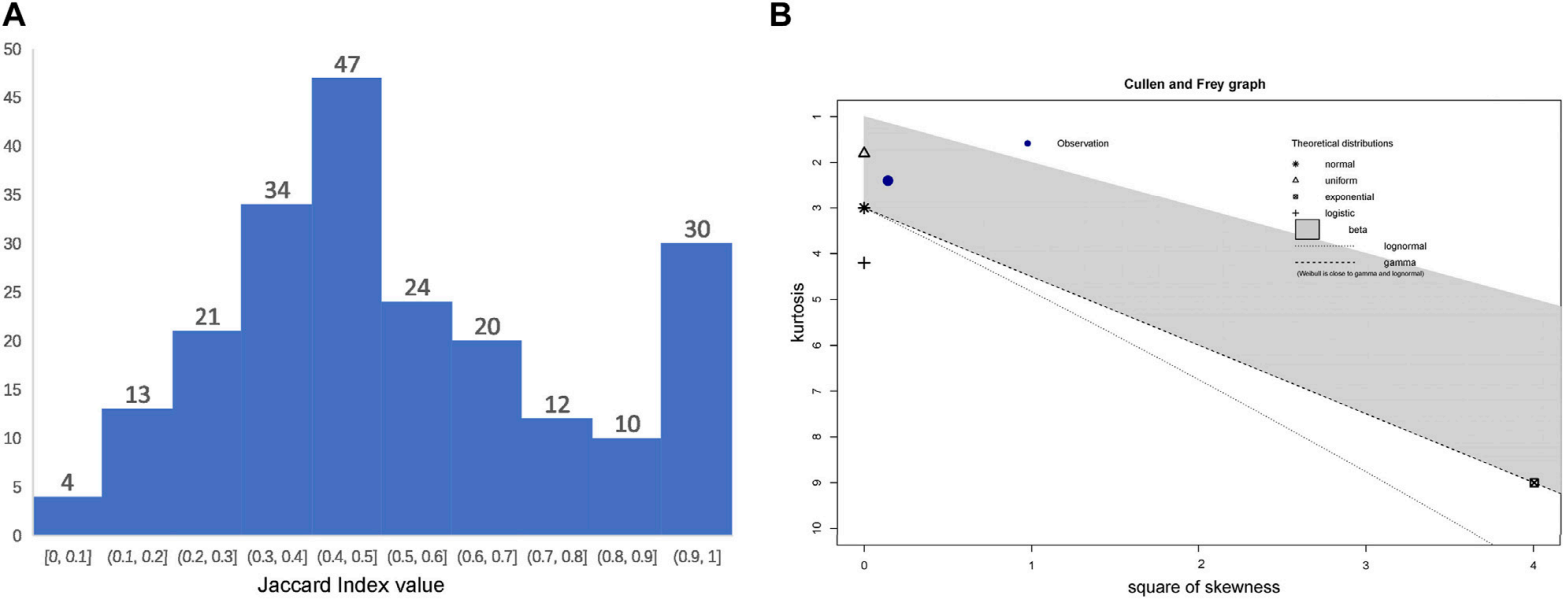
Distribution of Jaccard Index (JI) values of the botanical drugs from the same species. **(A)** the histogram; **(B)** a Cullen and Frey graph.

Different parts of 96 plant species are used as two or more drugs. For example, the root and the seed of *Abrus precatorius* L. are used as two drugs, separately; the ripe and unripe fruit of *Diospyros malabarica* (Desr.) Kostel are recorded as one drug but with different therapeutic uses, so we treat them as two single drugs. Volumes VIII and IX of API include 15 botanical drugs and their processed products (*i.e.*, powder, hydro-alcoholic extract, and water extract) respectively, and we treat these powders and extracts as the same drug with their original drug. In total, 238 single botanical drugs of API are sourced from these 96 species. The pairwise Jaccard Index (JI) was calculated to evaluate the similarity between every two drugs (Supplementary Material S3, note the ones sourced from the same species are highlighted). Figure 4 shows the distribution of pairwise JI values of the drugs from the same species.

Accordingly, the average value of all the 215 JI values is 0.54617, suggesting that drugs of the same species differ in their therapeutic uses. However, these values fit for beta distribution instead of Normal distribution. There are 30 JI values at the region (0.9–1.0], indicating that many of these drugs from the same species are still used highly similarly. For example, leaf, stem, aerial part and flower of *Dendrophthoe falcata* (L.f.) Ettingsh are recorded as four drugs separately, but they have very similar therapeutic uses; however, the therapeutic uses of its fruit are distinguishable. In other cases, the low JI values indicate the different therapeutic uses of drugs from the same species. For example, the seed and whole plant of *Datura metel* L. only have one common therapeutic use for PAR, and their JI value is 0.1. As is shown in Figure 4A, only four of these drugs are with distinct therapeutic uses, *viz.* the seed and whole plant of *D. metel* L., and the root and the stem of *Saccharum officinarum* L. As a result, the therapeutic uses of the drugs sourced from the same species may differ to different extent.

### Phylogenetic diversity and structure of the source plants of the single botanical drugs in API

Indices for phylogenetic diversity and structure of the source species of the single botanical drugs in API (Table 2) indicate the Ayurvedic medicinal plants for different therapeutic uses distribute evenly in the perspective of phylogeny. Faith’s phylogenetic diversity (PD) correlates with the numbers of species (Nsp) positively, fitting the formula PD = 44.836*Nsp +2,197.3 (*R* ^2^ = 0.9833) and this linear relationship hints that the phylogenetic distances between the species are even. This evenness is also supported by the similar Dp values which range from 110 to 142. Similarly, values of mean pairwise trait distance among taxa (MPD) also fluctuate slightly, while values of the mean distance to nearest neighbor trait distance (MNTD) are with relative larger fluctuation. Net Relatedness Index (NRI) and Nearest Taxon Index (NTI) are indices for indicating the significant clustering. Specifically, values below −1.96 indicate significant “over-dispersal” while values above 1.96 indicate significant “clustering.” As are shown in Table 2, there is no significant “over-dispersal” or “clustering” except for those of GAS and MET, which are clustering significantly.

**TABLE 2:** Indices for phylogenetic diversity and structure of the source plants of the single botanical drugs in API.

Site	Nsp	PD	Dp	MPD	NRI	MNTD	NTI
ALL	393	18765.34	131.88	264.43	0.9995	61.68	0.9995
AND	24	2480.24	117.39	244.98	0.6997	157.66	0.2298
ANT	63	5044.97	133.93	272.17	−0.5123	105.79	1.3870
CAR	201	11966.57	132.83	267.00	−0.4594	74.86	1.5728
DER	250	13495.03	130.95	262.95	0.2306	71.16	1.2967
EAR	31	3198.60	128.78	266.15	−0.0890	156.61	−0.2516
EYE	47	4184.65	125.91	257.28	0.3816	132.53	0.1500
GAS	293	15082.26	132.10	265.10	−0.1841	65.45	2.0526
GNR	255	13565.71	129.71	260.44	0.9152	71.98	0.7149
GYN	82	5978.06	129.91	263.03	0.1332	96.41	1.6847
IMM	93	7497.75	134.63	272.19	−0.6370	111.51	−0.5217
MET	227	12305.72	132.67	266.50	−0.3181	67.28	2.9931
NER	103	7643.13	135.95	274.56	−0.9384	106.09	−0.3789
ORA	45	4297.89	127.04	259.85	0.2454	146.78	−0.7199
PAR	134	9205.82	138.41	278.90	−1.5691	96.78	−0.4259
PED	12	1477.36	110.68	241.49	0.5930	166.16	0.8324
PSY	44	4191.30	137.91	282.23	−0.8910	140.39	−0.1611
RES	181	10411.27	132.77	267.02	−0.3242	77.24	1.6584
SKE	35	3692.30	142.50	293.39	−1.3157	141.90	0.3498
TRA	35	3205.85	118.51	243.99	0.9674	127.97	1.0970
URO	125	8299.06	125.44	252.91	1.1770	94.45	0.3384

Nsp, number of species; PD, faith’s phylogenetic diversity; Dp, diversity weighted by interspecific phylogenetic distances; MPD, mean pairwise trait distance among taxa; NRI, net Relatedness Index; MNTD, mean distance to nearest neighbor trait distance; NTI, nearest Taxon Index.

The algorithm “nodesig” in phylocom allows for finding the taxa of “hot nodes,” which were hypothesized to have higher potential for specific therapeutics. Accordingly, 172 species were found out in “hot nodes,” contributing 276 “hot nodes” hits for 20 of the therapeutic categories. Table 3 shows the number of these species by family and their hot nodes hits of each therapeutic category.

**TABLE 3:** Number of species in hot nodes by family and the hot nodes hits of each therapeutic category.

Family	Hot nodes hits^#^	Number of species in hot nodes	AND	ANT	CAR	DER	EAR	EYE	GNR	GYN	IMM	MET	NER	ORA	PAR	PSY	RES	SKE	TRA	URO
Moraceae	32	8	0	0	8	8	0	0	0	8	0	8	0	0	0	0	0	0	0	0
Apocynaceae	31	13	0	0	0	13	0	0	13	0	0	0	0	5	0	0	0	0	0	0
Poaceae	29	15	0	0	0	0	0	0	0	0	0	15	0	0	0	0	0	0	0	14
Fabaceae	22	18	18	0	0	0	0	2	0	0	0	0	0	0	0	2	0	0	0	0
Asteraceae	20	13	0	0	0	0	0	0	13	0	0	0	7	0	0	0	0	0	0	0
Apiaceae	19	11	0	0	0	0	0	0	11	0	0	0	5	0	0	0	0	0	3	0
Lamiaceae	18	7	0	4	0	0	0	0	0	0	0	0	0	0	4	3	0	0	3	4
Acanthaceae	14	7	0	0	0	0	7	0	0	0	7	0	0	0	0	0	0	0	0	0
Cucurbitaceae	11	11	0	0	0	0	0	0	0	0	0	11	0	0	0	0	0	0	0	0
Zingiberaceae	10	10	0	0	0	0	0	0	0	0	0	0	0	0	0	0	10	0	0	0
Pinaceae	6	4	0	0	0	0	0	0	0	0	0	0	0	0	4	0	0	2	0	0
Piperaceae	5	5	0	0	0	0	0	0	5	0	0	0	0	0	0	0	0	0	0	0
Convolvulaceae	5	5	0	0	0	0	0	0	0	0	0	0	5	0	0	0	0	0	0	0
Dipterocarpaceae	4	2	0	2	0	0	2	0	0	0	0	0	0	0	0	0	0	0	0	0
Cyperaceae	4	2	0	0	0	0	0	0	0	0	0	2	0	0	0	0	0	0	0	2
Typhaceae	4	2	0	0	0	0	0	0	0	0	0	2	0	0	0	0	0	0	0	2
Ebenaceae	4	2	0	0	0	0	0	0	0	0	0	0	0	0	0	0	0	2	2	0
Lauraceae	3	3	3	0	0	0	0	0	0	0	0	0	0	0	0	0	0	0	0	0
Meliaceae	3	3	0	3	0	0	0	0	0	0	0	0	0	0	0	0	0	0	0	0
Gentianaceae	3	3	0	0	0	3	0	0	0	0	0	0	0	0	0	0	0	0	0	0
Bignoniaceae	3	3	0	0	0	0	3	0	0	0	0	0	0	0	0	0	0	0	0	0
Aristolochiaceae	3	3	0	0	0	0	0	0	3	0	0	0	0	0	0	0	0	0	0	0
Pteridaceae	2	2	0	2	0	0	0	0	0	0	0	0	0	0	0	0	0	0	0	0
Brassicaceae	2	2	0	0	0	0	0	2	0	0	0	0	0	0	0	0	0	0	0	0
Cannabaceae	2	1	0	0	0	0	0	0	0	1	0	1	0	0	0	0	0	0	0	0
Rhamnaceae	2	2	0	0	0	0	0	0	0	0	0	2	0	0	0	0	0	0	0	0
Caprifoliaceae	2	2	0	0	0	0	0	0	0	0	0	0	0	0	0	2	0	0	0	0
Magnoliaceae	1	1	1	0	0	0	0	0	0	0	0	0	0	0	0	0	0	0	0	0
Simaroubaceae	1	1	0	1	0	0	0	0	0	0	0	0	0	0	0	0	0	0	0	0
Martyniaceae	1	1	0	0	0	0	1	0	0	0	0	0	0	0	0	0	0	0	0	0
Pedaliaceae	1	1	0	0	0	0	1	0	0	0	0	0	0	0	0	0	0	0	0	0
Verbenaceae	1	1	0	0	0	0	1	0	0	0	0	0	0	0	0	0	0	0	0	0
Loranthaceae	1	1	0	0	0	0	0	1	0	0	0	0	0	0	0	0	0	0	0	0
Santalaceae	1	1	0	0	0	0	0	1	0	0	0	0	0	0	0	0	0	0	0	0
Ulmaceae	1	1	0	0	0	0	0	0	0	0	0	1	0	0	0	0	0	0	0	0
Cupressaceae	1	1	0	0	0	0	0	0	0	0	0	0	0	0	1	0	0	0	0	0
Taxaceae	1	1	0	0	0	0	0	0	0	0	0	0	0	0	1	0	0	0	0	0
Marantaceae	1	1	0	0	0	0	0	0	0	0	0	0	0	0	0	0	1	0	0	0
Musaceae	1	1	0	0	0	0	0	0	0	0	0	0	0	0	0	0	1	0	0	0
Pontederiaceae	1	1	0	0	0	0	0	0	0	0	0	0	0	0	0	0	1	0	0	0
Total	276	172	22	12	8	24	15	6	45	9	7	42	17	5	10	7	13	4	8	22

# “Hot nodes” were found by phylocom 4.2, one species may be “hot nodes” of several therapeutic categories. A detailed list of these species in hot nodes is presented in Supplementary Material S4.

The results show many useful clues for the relationship between families and therapeutic uses. Eight species of Moraceae contributing the most hot nodes hits (32) to CAR, DER, GYN and MET, suggesting Moraceae’s potential functions in the above therapeutic uses; 13 species of Apocynaceae are found to be hot nodes of DER, GNR and ORA; almost all the hot nodes species of Poaceae are found to linked with MET and URO; 18 species of Fabaceae are with high potential for the therapeutic use of AND, which 2 of them are also for EYE. Several species are found to be related to more than one therapeutic use. For example, *Artocarpus heterophyllus* Lam. (Moraceae) is “hot” for CAR, DER, GYN and MET, *Coleus forskohlii* (Willd.) Briq. (Lamiaceae) for ANT, PAR, PSY and URO, *Ocimum tenuiflorum* L. (Lamiaceae) for ANT, PAR, PSY and URO, highlighting their multiple functions. With regards to the therapeutic categories, GNR and MET have the most hot nodes species (45 and 42 separately), this might because of the frequent occurrence rate of related diseases such as fever. Hot nodes species of GNR are commonly found in the Apocynaceae (13), Asteraceae (13), Apiaceae (11) and Piperaceae (5), while those for MET are from the Moraceae (8), Poaceae (15), Cucurbitaceae (11), Cyperaceae (2) and Typhaceae (2). Additionally, there are 13 families that have only one hot nodes species for one therapeutic category. It is worth noting that no hot taxon was found for GAS or PED, which can be accounted for based on different reasons: (a) There are 293 species (accounting for 74.5% of all the 393 species) with the therapeutic use for GAS, while the algorithm “nodesig” does not detect any “hot nodes,” hinting that no taxonomic preference for these species; (b) differently, only 12 species are used for PED, which is not sufficient for finding out the phylogenetic distribution pattern.

## Discussion

Ayurveda has provided medical services for people of South Asia for thousands of years. Currently, Ayurveda is embedded in the Indian national traditional medical system, and has been increasingly accepted globally since it offers a perspective on a “healthy lifestyle” instead of allopathy (Jaiswal and Williams, 2017). As a key reference for identification and quality control, part I of API records the most commonly used Ayurvedic single drugs. While it was reported that 70% of the Indian medicines are sourced from natural sources, and plant-derived drugs comprise 80% of the Ayurvedic medicines (Joshi et al., 2017; Sen and Chakraborty, 2017). Here, we find 621 single botanical drugs from all the 645 pharmacopeial monographs, and this high proportion highlights the importance of botanical drugs in Ayurveda.

The biological sources of the single botanical drugs are highly diverse. Previous studies have provided partial understanding on Ayurvedic medicines (Jia et al., 2012; Jaiswal et al., 2016). By systematizing the biological sources and indications of all the single botanical drugs in API and analysing their uses in terms of plant systematics, our study has made a vital step forward for a cross-cultural understanding of these medicinal knowledge system. It is expected that this fundamental information on Ayurvedic medical flora provided by the current study will foster the further development of Indian herbal resources.

The application of comparative phylogeny allows for refining the high potential taxa for specific therapeutic uses. This method has been used for comparative studies among medical floras and is found to be sufficient for predicting the plants with high medical value from a regional flora (Saslis-Lagoudakis et al., 2012; Lei et al., 2020). Differently, the present study focuses on the commonly used single botanical drugs without considering the regional flora, and the analysis is for refining of the traditional medicinal information. This is similar to the study on exploration of Chinese medical flora (Zaman et al., 2021). However, unpredicted results are also found. As a result, our directional results provide useful clues for bioprospecting of the botanical drugs. Our study also indicates that this method is a pragmatic tool for quantitative ethnobotanic studies, especially in compiling the traditional uses of a large number of plants with different levels of phylogenetic relationship.

The sharing of traditional medical knowledge and cross-cultural collaboration is essential in an era of globalization since health threats are a common challenge to all. However, without a cross-culturally usable or “standard” terminology transforming, the traditional health knowledge is difficult to be accepted cross-culturally or developed scientifically (Yao et al., 2021; Heinrich et al., 2022a; Heinrich et al., 2022b; Yao et al., 2023). While we clearly need a culture-specific expression of medical uses, as soon as such uses are to be compared cross-culturally, a standard terminology is essential. This study systematises the traditional herbal knowledge of Ayurveda using an etic view, which is expected to accelerate the global recognition of Ayurvedic botanical drugs.

## Conclusion

For the first time, the present work reports a systematic understanding on the single botanical drugs in API, which represent the medical flora of Ayurveda and are important global medical resources. We have clarified the single botanical drugs and their biological origins, as well as their standard therapeutic uses; additionally, the taxa with high potential of medical values for some specific therapeutic uses are predicted. Finally, we provide a ready-to-use medical ethnobotanical information on the Ayurvedic botanical drugs.

An increasing number of traditional, local herbal medicines are entering markets globally. The cross-cultural understanding on their traditional herbal knowledge is a core foundation for understanding such developments. Quantitative ethnobotanic methods are useful in understanding traditional herbal knowledge by providing standard outcomes which can form a basis for a cross-cultural comparison. Of course, such approaches are science- oriented (etic) and we do not address culture specific meaning aspects. The extending use of this methodology is expected to help in understanding the regional used traditional herbal medicines.

## Data Availability

The original contributions presented in the study are included in the article/Supplementary Material, further inquiries can be directed to the corresponding authors.
