# Supplementary material for: Single botanical drugs in the Ayurvedic Pharmacopoeia of India—A quantitative ethnobotanical analysis
Source: Front Pharmacol. 2023 May 11;14:1136446. doi: 10.3389/fphar.2023.1136446 (PMC10213908; doi:10.3389/fphar.2023.1136446)
Supplement: Supplementary file 1 [file DataSheet2.pdf]

S2. the phylogenetic tree of the source species of the single botanical drugs (inner part) and their therapeutic uses (outer part)

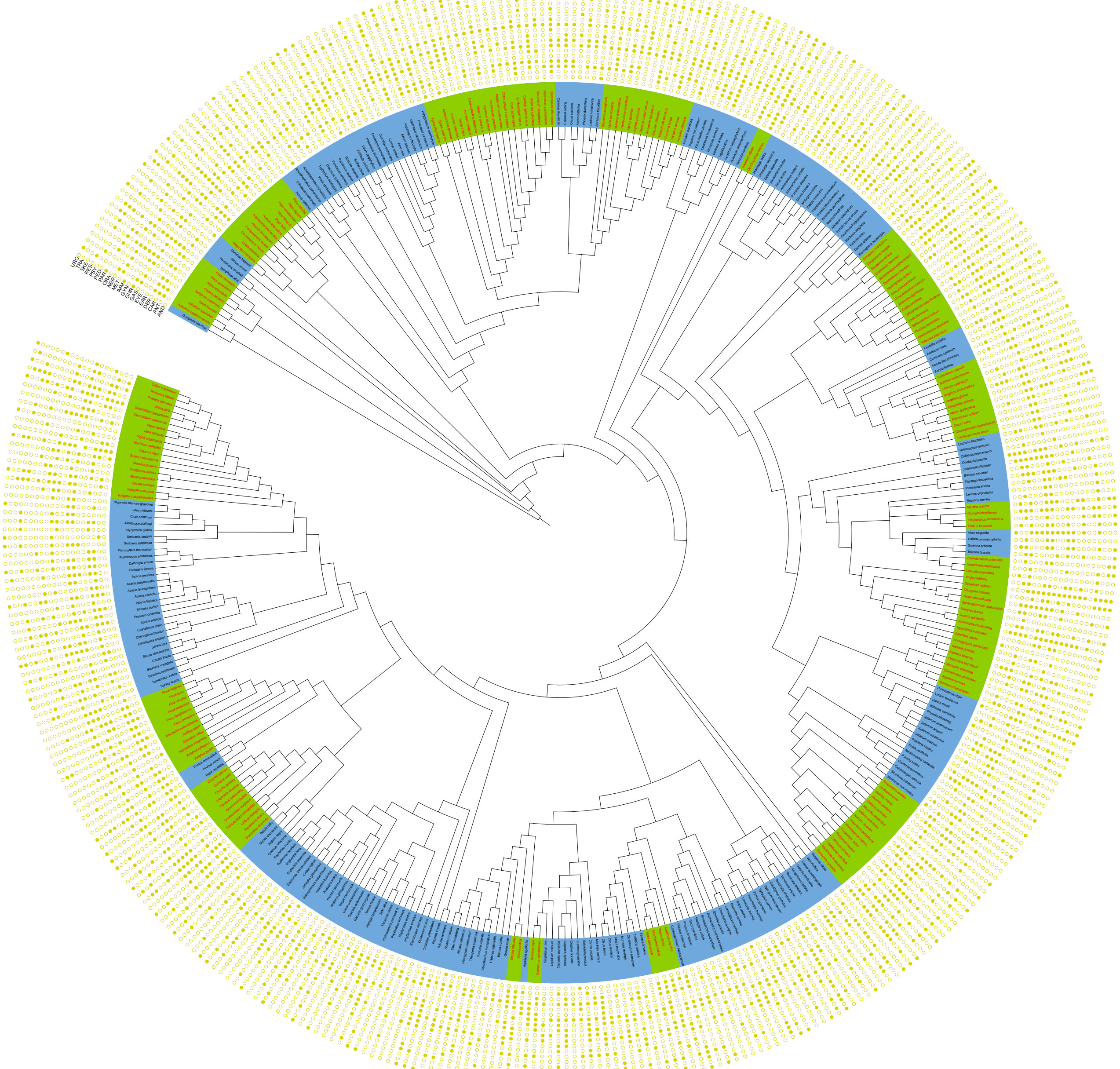

Note species of "hot nodes" for specific therapeutic uses are shown in red.
